# Dental Progress as Indicated in Current Events

**Published:** 1908-11-15

**Authors:** George C. Bowles


### DENTAL PROGRESS AS INDICATED IN CURRENT EVENTS.

BY GEORGE C. BOWLES, D. D. S.

Read before the Michigan First District Dental Society, October 8th, 1908.

Some observers think dentistry is moving backwards, but its literature both marks and indicates its progress. It has two main currents, the scientific, arising from the largely unappreciated and unrewarded labors of the few specially equipped explorers after the origin and cause of things; and the technical which is largely descriptive of methods and appliances. The average practitioner keeps tab on the advanced methods of constructing gold inlays or crowns and bridges, but gives too little heed to the theoretical or the scientific phase of his profession; both are essential to progress. There is no fear that the technical will be neglected; therefore, we shall deal largely with the scientific. We shall refer briefly to some of the more noteworthy productions of recent date, which may be more or less familiar to some, but are worth rehearsing in the connection we consider them at this time.

The cause of erosion, or the wasting of tooth structure, has engaged the labor of at least one of our greatest scientific workers for several years past. The work done by him in this field is stupendous, interesting, and may be of the greatest value in solving this perplexing problem. The results of two hundred experiments, requiring ten thousand hours of the time of Prof. W. D. Miller and his assistants, appearing in the January, February and March numbers of the *Dental Cosmos* for 1907, under the caption "Experiments and Observations on the Wasting of Tooth Tissue, Variously Designated as Erosion, Abrasion, Chemical Abrasion, Denudition, Etc." are well worth our study. In the

July *Cosmos*, appears another paper entitled, "Further Investigations of the Subject of Wasting." This is a monumental work, but exhaustive as it is, much is still left unsettled. Prof. Miller intended to continue his work on this interesting problem, but alas for mankind, that master mind has made its last gift to that widening human knowledge toward which he contributed so generously. While all the factors entering into the process of wasting, erosion, or abrasion, are not settled, Prof. Miller has shown conclusively that great damage results from coarse, gritty tooth powders, and the too vigorous use of the brush horizontally applied. With the tooth brush and the tooth powder bought in the open market, he was able, in a surprisingly short time, to reproduce, in teeth out of the mouth, the various phases of abrasion and wasting commonly found in it. We, as a profession, should give greater heed to this subject of dentifrices, tooth brushes, and the possibility of damage therefrom. Some of Prof. Miller's conclusions follow. See *Cosmos*, March 1907.

"Wasting of the teeth is for the most part a purely mechanical process (abrasion), in which and often the only factor concerned is the tooth brush in conjunction with the tooth-powder. While all powders or pastes containing pumice, cuttlefish bone, or oyster-shell are particularly injurious, excessive brushing with milder preparations may severelv abrade the dentin, and in course of years even the enamel. Abrasion may be readily produced artificially by the action of brush and powder."

"It has not been conclusively proved that wasting may occur, even in isolated cases, without the use of the toothbrush or some corresponding mechanical agent."

"Acids in general, especially in the strength in which they occur in the mouth, cannot produce wasting. They decalcify the tissue, rendering the enamel chalky and friable, and the dentin soft. Further than that their action does not go."

"As one who has been experimenting for the last four years on the subject of enamel erosion and abrasion, I should like to offer a few criticisms on Dr. Miller's detailed and suggestive work," writes Dr. Joseph Head, in the August 1907 *Cosmos*. From Dr. Head's paper, as also from his letter to the editor, and the editorial reply thereto, both in the April *Cosmos*, it would appear that even Dr. Miller, omniverous reader and indefatigable worker that he was, could not keep up with the rapid strides of science. In his experiments referred to above, he relied on the litmus test "as ordinarily employed in chemical laboratories" to determine the acidity, alkalinity, or neutrality of the saliva, and this, probably, has rendered questionable those of his observations, experiments and conclusions, that depended upon this test. Recent study has shown that in some cases saliva will turn blue litmus paper red, and at the same time turn red litmus paper blue, "in other words it exhibits what is known as the amphoteric reaction. This "curious phenomenon" is due to the presence of two salts, acid sodium phosphate, and basic sodium phosphate. "The fact that these two salts are incapable of chemically neutralizing each other is of the utmost importance in the study of the reaction of the saliva for the reason that the reaction of the oral fluids is a factor of fundamental importance in connection with the phenomenon of dental caries and likewise of dental erosion." (Kirk, *Cosmos*, April 1907.

Some of the possible errors into which the dependence on the litmus test led Dr. Miller, are pointed out by Dr. Head in the article referred to. Speaking of the inadequacy of litmus he says that a 1:20,000 solution of acid sodium phosphate will act more vigorously on a tooth than a 1:20, or a 1:2,000 solution, and yet it will neither turn blue litmus red, nor red litmus blue. On the other hand, 3 parts of a 1 per cent solution of acid sodium phosphate added to a 1 part of a 1 per cent solution of tribasic sodium phosphate will turn blue litmus red but enamel immersed in it for 18 days remained unharmed.

"At present we have to confess that we have no means of detecting minute quantities of acid in the saliva that may nevertheless, be sufficient to corrode the teeth; while on the other hand, acid may be plainly indicated and yet no harmful action result. It should be noted that the saliva, instead of being the cause of tooth decalcification, as some have supposed, seems on the contrary to be a valuable protection to the teeth against the invasion of corroding acids."

This opinion does not appear to be in entire accord with the findings of Drs. S. F. Acre, and J. E. Hinkins, as reported in a highly scientific paper "On Abnormally Acid Saliva" appearing in the *Dental Review*, April 1908. "The normal saliva is slightly alkaline. But there are many individuals who normally have acid saliva, the consistency and acidity of which vary remarkably with changes in health and habit. In such individuals general erosion of the teeth is one of the distinguishing features and the ravages of this malady may become so great that the teeth are eroded to the gum margins and only the roots are left. What is the nature of these acids? Are they organic or inorganic, or acid salts like sodium, di-hydrogen phosphate, or a mixture of all of these?" They may be produced in the following ways:

1. Acids can be generated by bacteria in the saliva after it has entered the mouth, bacteria having come into it from the mucous membrane or from the teeth.

2. Bacteria, aerobic and anaerobic, imbedded in the mucous membranes of the mouth, and lodged in or upon the teeth, can generate acids in situ from constituents of the saliva or from epithelium, which can then mix into the saliva and make it acid.

3. Aerobic and anaerobic bacteria, inhabiting carious places in the teeth, generate acids which may exude into the saliva.

4. Bacteria inhabiting the ducts leading into the mouth can generate acids from the constituents of the saliva or from the walls of the ducts, and this acid is carried into the mouth with the saliva. This may occur in all or only one of the many ducts.

5. The saliva can be secreted acid in the glands, because of a general acidosis and a tendency of all the secretions to be more or less acid. The acidity may be due, perhaps, to some abnormal action of the gland or to be decomposition of some easily hydrolyzed abnormal constituent of the saliva; the saliva then enters the mouth acid. This may take place in all or only some of the glands furnishing secretions for the mouth."

After a large number of most pains-taking, time-consuming and ingenious experiments the writers conclude that "it is evident that processes 1, 2, 3, and 4 cannot be greatly concerned in the formation of the acids found in the saliva of the individuals under investigation, that the saliva is probably secreted acid in the gland, and that bacteria has little or nothing to do with the acid production.

The determination of the nature of these acids must be left to the future, and other methods for isolating and identifying these acids and their salts must be established."

The authors used a very ingenious method for isolating the saliva from the parotid gland. . A device like the ordinary medicine dropper, but specially drawn and containing a fine platinum tube, was introduced into Steno's duct, and the uncontaminated saliva collected before it entered the mouth. In each case the secretions thus obtained was acid, in one more acid than the mixed saliva in the mouth. There is food for thought here for the prophylaxis practitioners. If teeth are rendered sensitive at their necks, in consequence of an acid saliva being poured upon them, how can we expect to modify this by any amount of polishing? Indeed, accepting their findings, the local treatment for this condition, the use of alkaline washes, powders, friction, polishing, etc., is about as promising as the effort to bail out the Mississippi river with a sieve. This investigation of Miller's should be taken up and pursued to completion in the interest of oral prophylaxis.

### PERICEMENTAL INFLAMMATION.

In the December 1907 *Cosmos*, Dr. Julio Endelman, has an instructive paper on "The Pathology of Pericemental Inflammation." The tissue changes occuring from the inception to the conclusion of the inflammatory process are followed step by step as they have been revealed by recent studies in cellular pathology and bacteriology aided by physiologic and pathologic chemistry. The article may well be referred to by any one wishing a clear and concise statement of the present understanding of this subject. Under the sub-heading "Remote Consequences" the author says: "Owing to the intimate relations of the teeth and soft tissues of the mouth with practically every organ of the body, it not infrequently happens that pericemental infections cause serious and at times fatal consequences. We have seen traced to this source, disorders of the intestinal tract leading to pernicious anemia and of the respiratory tract leading to purulent broncho-pneumonia. Septicemia and pyemia ending in death have also been caused by pericemental infections, likewise disturbances of the eye, ear, nose, throat and antrum; necrosis, partial and complete, of the lower jaw, facial paralysis, mandibular trismus, etc." He follows with a record of twenty-one serious clinical cases, two of them resulting fatally, all due to alveolar abscesses. In conclusion he says: "In the discussion of the remote consequences of pericemental infection, the writer's purpose has been simply to call attention, with the assistance of clinical observations, to the necessity of viewing pericemental infection in the light of the serious disturbance it really is, and not mainly as a purely local disorder."

### ALVEOLAR INFECTION.

In the same number, and written with the same purpose, is a paper by Dr. George Mitchell, entitled "Alveolar Infections; Extraction vs. Retention." After reviewing the literature on the subject in which there is a general agreement that "healthy oral cavities and their adnexa are especially exempt from infectious processes following injuries, while in an oral cavity, which is septic from an abscessed tooth, traumatism without preliminary and after treatment is very dangerous on account of the possibility of extension of the suppuration to some distant part." The possibility of severe intoxications following suppuration of the gingivo-dental regions explains the necessity of combating by all means at our disposal any suppuration, whatever be its degree of intensity." He includes letters from Drs. G. V. Black, E. C. Kirk, G. V. I. Brown, R. Ottolengui, R. H. Hofheinz, and M. I. Schamberg, received in reply to the following questions:

1. What, from your experience and observation, is your opinion of the following statement: "There are infections following the removal of abscessed teeth. Patients die, and the cases are not reported: they are entered at our hospitals as cases of pneumonia, but they are cases of septic pneumonia, resulting from abscessed teeth." Dr. Robert T. Morris, *Dental Cosmos*, July 1904.

2. Do you, (or did you) ever hesitate to extract in cases of pericementitis and alveolar abscess, or any acute alveolar infection?

3. If so, in what instances and for what reasons?

4. Do you differentiate in extraction between pericemental or alveolar infections caused by colds, and those due to other causes?

5. Do you think there is any danger attending extractions at any stage of alveolar infection?

6. If so, what dangers, and for what reasons?

The following reply of Dr. Hofheinz, is very concise and embodies substantially the answers of all the others.

1. There is infinitely more danger in leaving an abscessed tooth in its socket too long than there can be in its removal.

2. I should never hesitate to extract in cases of pericementitis or alveolar abscess, providing I could not by other means restore the surrounding tissues to a normal condition.

3. Causes of all pus-formation should be well and carefully differentiated. If due to a local affection, cure can certainly be much easier obtained than if it were due to a systemic diathesis.

4. I can see no danger in extraction it means, in most cases simply the removal of the cause—the rational treatment of all disease. Septic pneumonia, embolic pneumonia, if at all due to abscessed teeth, is not due to the extraction, *per se*. It may be due to the fact that the teeth were not extracted in time, thus allowing the pus to be absorbed, and so produce a remote septic condition."

Dr. Black says in part in answer to the fifth question, "There is often danger of septicemia resulting from alveolar infection. I think that the extraction of the teeth in these conditions is the best way to avert the danger though it may not always be successful. There are quite a number of deaths occuring from infections in connection with alveolar abscesses that are not reported as such. Several have occurred in Chicago. In my own practice, death has been very narrowly averted in a considerable number of cases. The danger to life in alveolar abscess is not at all sufficiently recognized."

In conclusion the writer calls attention to the necessity for the thorough sterilization of the mouth before the extraction of an abscessed tooth, also the complete sterilization of the mouth after the extraction of an abscessed tooth, also the complete sterilization of all instruments employed in the operation. The wound should be treated antiseptically until all possibility of infection is passed. "To neglect this important operation is criminal malpractice."

Because the vast majority of cases of alveolar abscess terminate without recognized untoward results, I believe we have come to look upon this condition all too lightly. In its treatment it rarely occurs to any of us, if indeed it is known to many, how really serious the matter is. The facts should awaken us from our slip-shod apathetic methods to an acute realization of all the possibilities. May we not all pause and reflect on our ability to recognize, avert, or combat the possibly grave consequences which at any moment may confront us? Let us be assured that in future our treatment of pulpless teeth and the possibility of subsequent abscess will be miminized by the most conscientious and intelligent effort of which we are capable. We should examine the mouths of our patients and remove every wreck of a tooth beyond the probability of conservation. The common advice, "let it stay until it makes trouble" should at once be corrected to "let it be removed before it makes trouble."

ORAL PROPHYLAXIS

The dominant note in dentistry to-day is "Prophylaxis," it has a broad field for operation in this direction. Perhaps the assertion that prophylaxis is the dominent note in dentistry to-day, should be modified by the statement, that at least it should be, and in reality is rapidly so becoming. At present it is certainly a lusty infant, and has already shown its possibilities. Its ardent advocates believe it is destined to revolutionize future dental practice.

The indications are many however that the prophylaxis idea is extending far beyond the boundaries laid down by its original advocate, Dr. D. D. Smith. The crusade of the orangewood point is doing, and will continue to do, a good work; but it is an adjunct, not a finality. Few are satisfied that either dental caries or Pyorrhea alveolaris are wholly filth diseases. Both complaints are found in mouths well cared for, and often the least cared for mouths are immune. To find and control the underlying cause, is the task now engaging the efforts of some of our foremost investigators.

In this connection the report of Dr. Immanual Muntz, chairman of the committee of scientific research, for the Dental Society of the state of New York, *Cosmos* March 1908, page 263, is very interesting. The work of the committee is a continuation of the investigations begun four years ago by a former committee consisting of Drs. Frank

W. Low and J. Wright Beach, of Buffalo, to determine what chemical constituents of the salivia influences immunity to decay. A more detailed account of this work, and the conclusions drawn therefrom, appears in a paper entitled, "The Saliva and Tooth Decay" by Dr. Beach, *Cosmos*, May 1908.

Dr. Beach quotes from Dr. Low as follows: "Beginning my labors in the line of investigation to determine, if possible, what chemical constituents of the saliva were most persistently abundant in the mouths of individuals having remarkably poor teeth, I found none that were invariably present. Failing in my quest, I began, to investigate the saliva of individuals having remarkably good teeth. Then it was that I made, what I now believe, to be to dentistry an important discovery—namely, the most invariable presence of generous provings of potassium sulfocyanate.

I have examined the saliva of several hundred individuals. Among those having remarkably poor teeth I have found potassium sulfocyanate conspicuous by its absence. On the other hand with but few exceptions, I never failed to find abundant potassium sulfocyanate in the mouths where remarkably good teeth were exhibited. The bacteria of decay find lodgement wherever there is roughened and defective enamel. According to Black, "Whenever decay occurs elsewhere upon the teeth it is because the bacteria themselves, under favorable conditions, have the ability to form small films or plaques of a gelatinoid character glued upon some accessible surface of the tooth where they remain undisturbed for a sufficient time to allow the bacteria embedded in them to corrode the enamel surface." The theory that accounts for absence of these plagues in the saliva of individuals who have marked provings of potassium sulfocyanate rests on the fact that potassium sulfocyanate is a powerful solvent of all gelatinous substances.

"Experiments which I have personally conducted have proved—(1.) That saliva from the mouths of persons having an abundance of potassium sulfocyanate will dissolve gelatine to double the quantity of such as have no potassium sulfocyanate. (2.) That during a considerable immunity from decay the saliva shows strong potassium sulfocyanate provings; while in the same mouth during a considerable period of rapid decay the saliva contained no potassium sulfocyanate. (3.) That in a mouth where decay was rapidly progressing, and the saliva showed no potassium sulfocyanate, the administration, by the stomach, of that compound, one half grain per diem resulted in the abundant presence of potassium sulfocyanate, making the saliva resemble in appearance that from other mouths where no decay was present." In this connection I would especially call attention to this statement by the author of this paper: "The tincture of iron chlorid as prescribed by physicians will neutralize the action of potassium sulfocyanate in the saliva."

This paper and its discussion should receive a careful reading by all interested in general oral prophylaxis, as should also a paper by Dr. Edward C. Kirk, entitled "The Nutritional Question as Related to Dental Pathology" *Dental Brief* October, 1907. In this paper the composition of the saliva, in its relation to caries, and other dental pathologic conditions, is carefully considered. Dr. Kirk states that, "The composition of the mixed saliva changes from time to time, and that it is an index of the nutritional state at any given time, and that the nutritional state resulting from a given food habit is an important factor, if not the most important factor, in the problem of susceptibility or immunity to dental caries." Therefore before our prophylactic measures will really prevent "We must learn to so change the composition of the body juices as to develop a maximum of vital resistance to disease invasion, on the principle that good health is the best prophylactic against disease."

GOLD INLAYS.

Just now the gold inlay is having its inning, and the rooters are making such a din that scarcely any thing else can be heard. It is interesting to note the revolution Dr. Taggart has wrought in current dental literature, by the introduction of the cast inlay method. Up to within a month or two the magazines have been literally filled with the thousand and one best ways of making gold inlays by the matrix method. With his wax model and casting device Dr. Taggart has with one stroke, rendered every thing previously written on the subject of gold inlays, almost valueless, save as history. This, of course, does not apply to cavity preparation, which for both methods remains the same. To-day there are as many best devices for reproducing the wax model as a year ago there were ways of making and filling the matrix.

In an excellent paper on this subject read before the Second District Dental Society, of New York, and appearing in the April 1908, *Items of Interest*, Dr. Taggart, in closing his discussion, said, "When I come before you, and hear words from men I never saw before, praising the method and the system, and saying they had attained more beautiful results than they ever had before, I feel that I am abundantly repaid. There have been several attempts to refer to the commercial side; but the commercial end has never entered my head. It has been how much could I do for humanity and my brothers." Alike, by these most generous and exalted sentiments and his revolutionary invention Dr. Wm. H. Taggart has rendered dentistry his everlasting debtor.

EXPANSION AND CONTRACTION OF METALS.

Growing out of Dr. Taggart's contribution is a series of investigations conducted by Dr. Weston A. Price, of Cleveland, into the hitherto unexplored realm of the contraction of metals used in dentistry, "on cooling from their melting or freezing point to ordinary temperature." His paper, "The Laws of Determining Casting or Fusing

Results Their Control and a New and Rational Technique." *The Items of Interest*, for May, 1908, contains data invaluable in the perfecting of this new art. By a specially constructed instrument capable of measuring to the hundredth thousandth of an inch, connected with an electric heating device and a pyrometer, Dr. Price has determined, for instance, that 24k gold at atmospheric pressure contracts on cooling from the melting point, a trifle over twenty-two thousandth of an inch per linear inch, but the contraction decreases with the increase of pressure, and at a pressure of 5½ lbs, the contraction is reduced to thirteen thousandth or nearly one half, and concludes that "to secure the least contraction we must use as high pressure as possible without producing distortion of the investment." With sixteen pounds pressure, the actual pressure on an inlay ⅛ of an inch square, is less than ⅛ of a pound. Inlays cast at low pressure in an investment mixed soft and slowly dried out, contracts over forty thousandths, or over four percent. The contraction of such investment is as much as twenty-five thousandths." All the investments have a very large total contraction on cooling. To overcome the contraction of the gold, the investing material should expand fifteen thousandths at not to exceed 700 degrees, at which temperature all are very far short, the best have only half expansion enough and these having the most, have so little strength, that high pressure is likely to distort them. These defects the author believes he has overcome by a technique which he describes and an "artificial stone," a modified silicate cement, capable of resisting any required heat and pressure, from which he makes a cast in which the wax is formed. This artificial stone cast, containing the wax model, is invested, and the gold under high pressure forced into it. The resulting inlay from being cast in an investment the cavity aspect of which is as hard as stone and which expands on heating almost enough to compensate for the contraction of the gold, is well nigh perfect.

While Dr. Price fully realizes that he probably could turn the control of the composition and manufacture of this model material to considerable financial gain, it would be poor compensation for midnight oil,compared with giving it unhampered and as quickly as possible to the profession and humanity, whom he believes will be saved more suffering and discomfort through its means than the writer could relieve in a score of life times binding up wounds." This has a splendid ring and again demonstrates the difference between the competitive warfare dominating the commercial world and the co-operative spirit, the animating ideal of the professional calling. The benefits of such devotion and self sacrifice on the part of our leaders, accrues to the all in the profession and should render every man capable of having a spark of appreciation aroused within him, willing and eager to contribute all that within him lies, for the further establishing of our calling upon the broadest professional lines.

### NITROUS OXIDE AND OXYGEN.

Of great interest to those administrating nitrous oxide is a paper in the November, 1908, *Cosmos*, by Dr. C. K. Teter, entitled "Nitrous Oxide and Oxygon; Its possibilities as a General Anesthetic." He says: "Nitrous Oxide is not only an anesthetic but is an asphyxiant as well. If we can remove the latter quality we have an ideal anesthetic agent, as nitrous oxide is practically non-toxic and there is no agent known that is capable of producing narcosis with so little constitutional disturbance."

Because of this asphyxial element, Dr. Teter does not regard the administration of nitrous oxide safe when administered without a corrective. In this view he is confirmed by a most interesting observation he was enabled to make on the physical appearance of the brain, a portion of the skull having been removed, while the patient was under the influence of nitrous oxide, alone, and in combination with oxygen. To quote, "It is interesting as well as instructive to observe the effect of nitrous oxide and oxygen upon the brain and to note the difference when nitrous oxide is administered with and without oxygen. When the nitrous oxide is administered alone we find that as soon as the asphyxial element begins to enter into the procedure, the brain looses its natural pinkish color and turns more or less gradually to a dark purple. If the administration be continued without air or oxygen, it will take on an appearance resembling that of stagnant blood. As this discoloration progresses there is a dilitation of the brain, and the greater the discoloration, the greater the dilatation, so that it will protrude through an opening in the skull. One can imagine what this would mean to a patient with a myasthenic heart, or one with apoplectic tendency. This accounts for the headache sometimes complained of after the administration of an anesthetic. The condition of the brain is altogether different when a patient is anesthetized with oxygen in combination with nitrous oxide. On increasing the oxygen to almost one half of the mixture, it was but a few seconds, not more than nine or ten, until the brain assumed its natural color and returned to its normal position. Citing one case from among six hundred, in which Dr. Teter administered nitrous oxide and oxygen, for major surgical operations, he says: "The patient was under the influence of nitrous oxide and oxygen, without one breath of air, for nearly two hours and forty-eight minutes. Nearly six hundred gallons of nitrous oxide and eighty gallons of oxygen were used. Upon the completion of the operation, the anesthetic was withdrawn and the patient regained all her mental faculties within one minute. There was very little shock from the procedure, nausea and other post-anesthetic complications were entirely absent. Nitrous oxide and oxygen may now be safely employed to render all dental operations painless and the prediction is made by Dr. Teter that "The day is approaching when it will be absolutely necessary for us to do painless dentistry."

The intimate relationship of the anterior superior dental nerve with the nasal mucous membrane satisfactorily explains the anesthesia produced in the anterior teeth by the insertion of a tampon of cotton saturated with a cocaine solution in the nasal fossa. "The technique of anesthesia of the upper incisors by the method under discussion, is very simple, consisting in the introduction into the anterior region of the nasal fossa, of a tampon of absorbent cotton of the size and shape of an ordinary almond, saturated with a 10% solution of cocaine, to which a small proportion of adrenalin-chloride may be added, if desired. The cotton tampon should be placed beyond the cutaneous border of the nostril in close proximity to the anterior edge of the inferior turbinate." While the tampon is in the nose the patient should sit erect to avoid the possibility of some of the solution escaping through the posterior nares and gaining the deeper structures. As a rule anesthesia is not evident before twenty minutes after the insertion of the tampon and it reaches its greatest intensity in about thirty minutes. Occasionally the incisors and canine of the opposite side are also anesthetized. *Cosmos* February, 1908, page 180.

### ELECTRIC SLEEP.

On page 1215 of November *Cosmos*, 1907, reference is made to "Electric Sleep," a name given by Prof. Leduc, of Nantes, to a condition comparable to chloroform narcosis, produced by the action on the brain of an intermittent current of electricity of low tention, six volts, generated by the means of a source of continuous current and of a specially constructed form of interrupter. This "sleep," in which the subject lies without any power of sensation or voluntary motion, may be maintained for hours. "A remarkable fact is that when the flow of current is interrupted, the subject awakes immediately, without any of the after effects that follow chloroform narcosis." Let us trust that this "electric sleep" is not a pipe dream and that it may work a revolution in present methods of painless (?) dentistry, equal to that wrought by the cast inlay for the operation of gold fillings.

Some valuable and practical information on the subject of anesthetics has been brought together in a paper by Henry H. Boom, M.D. *Items of Interest,* October 1907. Cocain is particularly well considered.

### ORTHODONTIA.

While orthodontia has become a specialty and is passing more and more out of the hands of the general practitioner a campaign of education is required to instill a few of the fundamental precepts of orthodontia into the working habit of the general practitioner, as much of the specialists' time is employed in correcting the deformities the general practitioner should have averted. The constant recurrence of the word "mutilation" in the papers and discussions on this subject is not to our credit. The class of people who are willing to pay for the correction of irregularities, as a rule, have a family dentist and would be willing to pay him for the proper care of their children's teeth, did he, himself, appreciate the necessity, and inform the parents of it. Says Dr. Elliot R. Carpenter, *Dental Review,* April 1908. "I believe fully fifty percent of malocclusion is due to the extraction of the first permanent molar after patients attain twelve years of age."

### AMALGAM ALLOYS.

In a paper read before the National Dental Association, and appearing in the February *Cosmos,* Dr. Marcus L. Ward, shows that the best dental alloys, those carefully compounded after the Black formula:

| | | | | |
|---|---|---|---|---|
| Silver | 65 | to | 68% | |
| Tin | 26 | to | 28% | |
| Copper | 3 | to | 5% | |
| Zinc | 1 | to | 2½% | |

Both before and after amalgation possessed very unstable qualities. When freshly cut such an alloy requires a larger percent of the mercury to put it into solution, or to make a workable or plastic mass, its points of expansion are greater and its setting is quicker than an alloy that has been properly annealed or aged. A number of fillings $\frac{1}{8}$ inch in diameter and $\frac{1}{8}$ inch high made from five parts of such a freshly cut alloy, to seven parts of mercury, thoroughly amalgamated, and excess of mercury expressed, developed in forty-two days their maximum crushing strength of four hundred ninety-seven pounds. This crushing resistence gradually decreased until in less than two years it was one hundred and eighty-seven pounds. "But, in addition to the changes that take place in the movement that accompany the setting, the percentage of mercury and the time required to set the mass, the annealing produced by the alloy standing around causes a decided decline in strength. Alloys which resist five hundred pounds when new will often become so much annealed in one year that one hundred and fifty to two hundred pounds is the maximum strength which can be developed." Alloys kept at office temperature for a longer period than one year should not be used.

The papers referred to in this report will indicate the trend of current dental literature, better than any analysis the writer might make, they will also refute, it is believed, the contention of Dr. Talbot, that dentistry is going backward. If we set out to find the common place, the imperical, the rantings of the blind would be leaders of the blind, we can find them everywhere, within as well as without the domain of dentistry. Dentistry has much to forget and very much to learn, but it is moving forward, and we have no call to be ashamed of its progress.

DISCUSSION.

DR. M. C. WARD: Mr. President and members of the First District Dental Society: Owing to the time consumed in the reading of the paper, I shall cut short some of my discussion that I had prepared. I have enjoyed very much the paper, and one or two phases of it have particularly interested me.

I think the most of you are familiar with the results of Miller's work on erosion. The effects of acids and gritty tooth powders occupying the main part of his attention. After an enormous amount of work Miller had apparently solved the problem as to the cause of erosion. However, his tests, as thorough as they had been carried through, and after the careful study that he had put upon them, were questioned seriously by Dr. Head and Dr. Kirk. He had used the litmus test for saliva reactions, and from his mechanical experiments drew his conclusions that the tooth brushand powder alone had caused the erosion, and that acids had no part in it. His tests for acids have been seriously questioned by almost all the chemists who have read his articles. While the work Miller did was very suggestive and is a grand lesson for us as to the proper use of the tooth brush and tooth powder, we cannot say to-day that acids of the mouth have nothing to do with erosion.

If Miller's tests with litmus were correct and could be proven, he neglected one thing which has since his research been carried further on, and that is the restraining influence of the saliva upon acids. I would speak of this in this connection because it has been neglected by those practicing oral prophylaxis. It has been one of my beliefs that even though you have cared for all the lactic acid in the mouth of any individual at a given date, you may not have cared for it in all the future, because the next week it is different, and the saliva in this same individual has changed. One of the simplest tests we have in the dissolving of tooth structures in general is to note the changed condition of the saliva and its restraining influence upon the lactic acid at times. A one to 500 lactic acid solution will dissolve tooth structure readily, but a one to 500 solution of lactic acid and saliva will not destroy a tooth in five years. We have been taught that lactic acid was the cause of dental caries, but have not been taught what the action of saliva upon lactic acid is. I think the conclusion which we are to draw is that the coarse powder and the tooth brush, used too vigorously, is to be deprecated and at the same time we are not to say that lactic acid, as it is found in the mouth, has no part in the erosion.

Drs. Cook, Hinkins and Acre claim to have carried on a lot of scientific work and determined that the normal saliva in the average individual is alkaline—but there are enough individuals in which it appears acid. Their research led along the same line with Miller, and they endeavored, of course, to solve the question of erosion. Their main work was to find the cause of erosion. They have not discovered it, though they believe they are on the road.

I want to impose on your time for a moment, to read a part of an article written by Dr. Kirk, in the August, 1908, *Cosmos*, on the Constitutional Element in Certain Dental Disorders, for its bearing on this subject of erosion, and also on one other reference in the paper namely, Systemic Consequences of Infection from Pericemental Abscess.

"The time has passed when the disorders which fall within the province of the dentist for treatment can be regarded as without definite relationship to the vital processes of the body. The advances which have been made in our knowledge of the physiology and chemistry of nutrition and the more intimate processes of metabolism are bringing much light to bear upon the interpretation of those abnormal vital phenomena which we call disease. The whole domain of pathology, and especially of etiology, has been undergoing a process of evolution in which the point of view, and correspondingly the point of attack, is being rapidly changed from the uncertain position of empiricism to the secure basis of scientific certainty. This change in the domain of general medicine necessarily has its reflex in a similar evolution in dentistry. The study of dental disorders from the restricted limitations of their purely local manifestations while it has taught us much with regard to their direct and obvious features, has done but little for their rational therapeutics and less for their successful prevention. With all that dentistry has accomplished we are as yet lacking in a knowledge as to the causes of susceptibility to any of the characteristics and well recognized dental disorders or of the conditions which insure immunity from them, and until we shall have solved these questions satisfactorily we shall have made but little progress toward their prevention in the first instance or their permanent cure by treatment.

With the discovery of the germ origin of certain diseases worked out to a certainty by Koch, in 1882, as the result of the exact methods of scientific research devised by him, and later followed by the application of these same methods by Miller to the study of dental diseases, a stimulus was given to the study of bacterio-pathology which has been so fruitful of results that there is scarcely a disorder known to man that has not in some way had its causation traced to bacterial agencies. So profound has been the impression made by the study of the bacterial factor in disease causation that for the time being it would seem that bacteria and their disease producing power had obscured the entire pathological horizon—so much so, in fact, that for a time at least the problem of disease causation seemed to have resolved itself in principle into the comparatively simple matter of reaction to bacterial invasion.

As an outgrowth of the recognition and general acceptance of this principle, tremendous expansion of the field of research in bacterio-pathology has taken place, until what may be termed the mechanism of bacterial invasion and its relation to disease production has been very clearly worked out with regard to a long category of pathologic conditions, the origin of which was previously obscure.

### SUSCEPTIBILITY AND IMMUNITY.

In the study of bacterio-pathology it was quickly discovered that individuals differ in their relative powers of resistance to bacterial invasion, and that these differences of resistence varied from time to time in the same individual, so that in the study of disease causation it became necessary to determine not only how bacteria produce disease, but why, in certain instances, they failed to produce disease. In short, the problem of immunity as well as of susceptibility presented for solution before a clear understanding of disease causation could be reached. Investigations of this interesting phase of the question have occupied the attention of numerous observors for years, and have been productive of results among the most brilliant in pathological research as well as of great humanitarian value.

DR. WARD, Continuing: I think that all the work that is being done and that has been done and referred to by the essayist to-night has a bearing upon this one question, and that the question is not yet solved. Dr. Kirk takes up this one topic and I think he takes the stand that the real root of the evil has not been gotten at; that erosion is the same as other aflictions of the body, influenced largely by the diet and and habits of the individual. He goes on to show that the end products of the proteids are the urates, which deposit largely in the peridental membranc and the articular membranes; that as a result over-proteid feeding results in the deposit of urates in these membranes, that this gives rise to rheumatism, pyorrhea, and a number of such afflictions such as may result from the deposit of the urates. It is invariably necessary for a completê diagnosis that we study the conditions of the saliva, the saliva being one of the prominent indicators of systemic trouble. On the other hand, is pyorrhoea being caused by the feeding of proteids? Kirk says that the feeding of excessive carbohydrates results in the formation of carbonic acid, and that in time faulty elimination of proteids by the kidneys produces increased elimination in other secretive organs of these products. In this way the salivary ducts may produce an acid saliva which may be a cause of erosion and interstitial gingivitis, and even pyorrhea may be promoted by some such faulty metabolism and vitiated glandular secretions. His treatment I am not prepared to discuss; I simply speak of this as a matter of interest in reference to the first part of the essayist's paper. I hope all of you will read Dr. Kirk's article, it may give us a better understanding of the part systemic influences play in pyorrhea and help us to apply our local treatment more intelligently. It will not supplant prophylaxis, but I think it is a good thing to keep in mind in any treatment.

Another part of the paper I should like to say a word about. I would also place considerable emphasis on the article by Dr. Teeter in regard to the use of oxygen with nitrous oxide, because to day it has been nearly discredited by the majority of dentists. Many have found some other apparatus that is easier to handle, or some other thing which serves the same puipose; but to my mind, they do not. Many have been unskilled in gas administration and have thought that the duration of the anesthesia was too short. I believe that for all such it would be well to read carefully Dr. Teeter's article. He claims, and I am confident he can carry out all his claims, that the true anesthetic properties possessed by other anesthetics can be secured by nitrous oxygen when used with oxygen. He has done operations of two or three hours, and the patient could talk to you in a minute or two after the anesthetic was withdrawn. If we could do this there is a decided advantage, and from my own experience it can be done in many other operations as well as on the teeth.

Dr. D. M. Graham: The one part which appealed especially to me was that which dealt with the role played by Potassium Sulphocyanate in the prevention of caries. Heretofore we have pointed with a good deal of pride to our restorations in gold and porcelain, but when we are asked the question what have we done toward preventing caries, or towards preventive dentistry, we are compelled to hang our heads in shame. Very little, indeed, has been done in the direction of preventive dentistry, prophylaxis possibly excepted, and while I am a believer in oral prophylaxis, in a large sense, I do believe that prophylaxis will, to a considerable extent, prevent caries, and I believe that a tooth that is kept perfectly clean will never decay, yet, how is it possible for us to keep the teeth perfectly clean? I have been ashamed more than once of being compelled to go to my card case and consult my records to know whether I had cleaned a patient's teeth only a fews days previously. We cannot always tell by their appearance whether we have cleaned a patient's teeth 24 hours before, and even if we have cleaned them 24 hours before, we are not sure that they are clean 24 minutes afterwards. So, if a perfectly healthy and cleaned tooth will not remain immune to carries, prophylaxis is not going to be a panacea for the prevention of caries. The great question with me is, just what will prevent caries, will this potassium sulphocyanate? So far as I am concerned myself, I am somewhat pessimistic regarding it, because so many theories have been advanced, in so many directions, and after the test of time they have all fallen down. I hope that this new suggestion will be of some service, it is very interesting indeed. I have made some of these tests and they have shown up satisfactorily. I have proven to myself in a npmber of cases that mouths comparatively immune to caries will show this test for potassium sulphocyanate, and in some mouths where caries is rampant I have found almost a total absence of sulphocyanate, and I am ready to raise the question whether this is the cause or the accompaniment. I should like to say that it is not possible for the general practitioner to prove this. I do not think it is possible for myself or any ordinary or busy practitioner to undertake these tests, and to carry them out carefully and conclusively enough to establish the fact. I believe this is a question that should be undertaken by a scientifically educated man, and carried along for a number of years, with sufficient data to prove beyond per-adventure that so and so is right; otherwise those of us who attempt this work hap-hazardly and indefinitely are simply adding to the work of the scientific man. The scientific men tell us, over and over again, that the greater part of their time is taken up in disproving theories advanced, and it seems to me that dentistry is of sufficient importance that it should support a scientific council, an investigating council of some kind, and such questions as this should come before it and should be solved by it; at least attempt at solution should be aided by scientific bodies of reliable men, whose findings could not be questioned.

All research work is characterized by an effort to discover, the exact nature of the more intimate features of the problem the forces or mechanism, physical or chemical or both, as it may be, by which the healthy body defends itself from attacks of pathogenic bacteria. Little by littlle the underlying facts of the phenomena of defense are being brought outwith every prospect that in its main principles the problem will be solved in the same way that we may, I think, fairly say that the modus of bacterial invasion and bacterial action in disease causation has been already solved. But whatever may be the terms in which the factor of bodily defense against bacterial invasion shall be finally expressed, or whether the formula for the opsonic index is to be written in the concrete symbols of a chemical entity, it is reasonably certain even now that what we call good health, the resultant of that complexus of all the factors entering into normal nutrition, the "harmonious adaptation of internal relations to external relations" as expressed by Herbert Spencer, is the condition in which the factor of defense against disease invasion—in a word, immunity—has its highest expression of efficiency.

It is not assumed by any means that the normally healthy individual is immune to the attacks of all pathogenic organisms, for in this as in all biologic questions the element of relativity is a controlling factor. It is, however, true that the liability to disease infection stands in an inverse ratio to the health status of the individual as measured by the nutritional standard, those with impaired nutrition manifesting a degree of susceptibility in proportion to their deficiency of health or degree of malnutrition. With respect to many cases of invasion by disease-producing bacteria in those instances where the defensive forces of the body have become impaired through malnutrition in any of its varied manifestations, we may regard such instances as terminal infections in the same sense that we speak of croupous pneumonia as a sequence of pathological dentition, or of acute miliary tuberculosis following Bright's disease as terminal infections. It is from this point of view that I think we are justified in considering certain dental disorders, more particularly that group of destructive inflammatory processes affecting the retentive structures of the teeth which we designate collectively as pyorrhea alveolaris, interstitial gingivitis, Riggs' disease, etc.

### THE PREDISPOSING ELEMENT.

It has long been observed that these alveolar lesions are manifested in individuals below the normal standard of health, those in whom errors of nutrition in varying degree are evident. But while the clinical fact of the coincident malnutrition has been noted, the tendency has been to attribute the depressed state of health to the alveolar infection rather than to regard the local alveolar disease as an expression of a general nutritional fault manifested as a terminal infection. It is undoubtedly true that the alveolar disease in its turn contributes to the general disturbance of nutrition by becoming a focus for the production of poisonous substances which, when carried into the general circulation, increase the total toxemia and intensify the general nutritional disturbance; in short, a *circulus vitiosus* is established which, unless corrected, gradually makes greater and greater inroads upon the health of the individual so affected. What I desire to emphasize, however, is that in the first instance the invasion of the alveolar structures was made possible by a lowering of the standard of vital resistance of the tissues of the individual by reason of some pre-existing nutritional fault, otherwise the infection of the alveolar structures would not have occurred; that is to say, the malnutrition stands to the alveolar infection in the relation of a predisposing cause. Unless we adopt this view we are without a basis of explanation for the fact that many people escape alveolar disease, for certainly their immunity cannot be explained upon the hypothesis of avoidance of contact with the particular pus-producing bacteria which has been found commonly associated with pyorrhea cases. If we admit the predisposing constitutional factor, it is evident that the therapeutics of this group of disorders must also include careful attention to those systemic states in which susceptibility to this disease is markedly evident, if we are to entertain the hope of intelligently and permanently curing the disorder.

Another strictly dental lesion which, from the point of view of your essayist, is directly traceable to systemic disturbances resulting from general malnutrition is chemical erosion of the teeth. I have elsewhere and at various times called attention to the fact that in erosion cases an acid condition of the mucous secretions of the buccal mucous follicles is clearly manifest, and further that the examination of a large number of specimens of both saliva and of buccal mucus taken from typical erosion cases has shown the presence of the acid phosphates of sodium and of calcium as a characteristic constituent of the fluids in question. Laboratory experiments amply demonstrate that both of those acid phosphates rapidly dissolve enamel, hence I am personally convinced that chemical erosion of the constitutional type is mainly due to the action of the acid phosphates, and that undoubtedly these salts when found in the oral fluids are evidence of a general nutritional fault. I should perhaps express my view somewhat more accurately if I said that they are evidence of a characteristic nutritional fault.

I have selected these two apparently distinct lesions, pyorrhea and erosion, as examples of dental disorders, having marked systemic relationship, but I would go farther and express the view that they have their origin in a fault of nutrition which in principle is the same for both, although its ultimate local expressions as erosion and as pyorrhea bear no clinical resemblance to each other.

It is a significant fact that the two disorders are frequently found in the same mouth—erosion in the upper denture and pyorrhea in the lower. I would not be understood as stating that both erosion and pyorrhea in an active state are to be found in the mouth of the same individual, but all have undoubtedly noticed instances of eroded teeth in the mouth of an individual some of whose other teeth have been attacked by alveolar disease.

Examination of the food habits of a large number of cases of persons suffering from chemical erosion of the teeth and of those having alveolar disease of a constitutional type has furnished me with personally convincing evidence that the local conditions are secondary to a predisposing factor of faulty nutrition due to errors of food habits, errors particularly of the kind and amount of food ingested with regard to the oxidizing power of the individual in each instance. And further, that while the general fault of both types is that of relatively deficient oxidizing power, the erosion cases are characterized as excessive carbohydrate-eaters, while those who were of the pyorrhetic class are victims of underoxidized proteid feeding. I fully anticipate the usual attitude of destructive criticism which the utterance of so broad a generalization is destined to call forth, and so I hasten to explain that my general statement is to be taken only in a relative and by no means in an absolute sense.

Dr. R. W. Bunting: The subjects are so many and important one does not know where to begin, nor where to end. Some of the points brought up by the essayist in the paper are of vital interest to all of us. This point in the paper attracted me especially, the sulfocyanate theory. In Dr. Black's new book he speaks of this work, saying that it has not been completed so that we can say anything as to the ultimate results of the research; but he states that from preliminary reports given out, that the precentages as shown bycareful examinations are very high. One particular feature is that the saliva which comes from the immune cases, has the highest percentages of sulphocyanate and on application to the mucoid plaques taken from the mouths of susceptible patients, it will be seen that this immune saliva will dissolve the bacterial plaques very readily, or break them up and entirely disintegrate them. Now we think we know, we believe at least, that caries is inaugurated and carried on, at least in the early stages, by means of a vitalizing membrane, which is a kind of mucoid, covering which protects the bacteria on the tooth's side from the saliva and agencies which would tend to wash them away, and, acting as a vitalizing membrane, bring in the carbo-hydrates from the outside to combine with or nourish the germs which form lactic acid, which produces decalcification. If it is possible that it can be shown conclusively that the sulphocyanate saliva will destroy these patches.

As to whether the raising the sulphocyanate index of the saliva by administering this drug internally is of any virtue, I do not know. I have not read enough to know whether this is proven conclusively or not.

One point seemed to be brought out very strongly in Dr. Kirk's article, as quoted by Dr Ward, and which I have read a number of times, carefully and with great interest, and the point which impressed me very much as being uppermost in Dr. Kirk's mind is his claim that it cannot be overlooked that many of our dental lesions, such as erosions, and pyorrhoea, must depend to a considerable extent upon the general nutritional conditions. If he has the right idea, if his conclusions are so and his observations are true, and we really do have general nutritional disturbances which play a part in erosion and in pyorrhea, it seems to me that in every case of pyorrhea we have three methods by which we may combat the disease. First, in regard to nutritional causes, the ultimate cause of which must be in the liver, where the urates and uric acids are formed, and which we are told should be eliminated by the kidney. In cases of nutritional disturbances, where the insoluble urates get into the blood, they travel naturally by the blood stream to the joints or the pericemental membrane. The blood streams, then, may constitute our second opportunity in scientific treatment by intercepting or correcting this condition.

Thirdly, the inflammation caused by these urates in the pericemental membrane where they are deposited is the third opportunity for practical treatment to be applied locally.

Now in the first place, if we possess the skill we can treat nutritional disturbance systemically and eradicate the disturbed function, that is to say, we can stop the course of the disease by removing the first cause in the liver and the kidneys, and correct the excess in acidity or alkalinity of the blood. Or we may, by the use of blood purifyers, kill it in the blood. After it has finally been deposited, and becomes localized in the gum, we can scrape it off the roots of the teeth and by systematic prophylaxis efforts, keep off future deposits, and make local conditions such as to favor resolution and a cure of the localized manifestations of the disease at least. If it is possible, it seems to me that it is much more reasonable to use the third together with the first of these remedies, that is to say, first take away what is deposited and then go back to the beginning and see that no more encouragement is provided than is absolutely necessary.

Dr. C. P. Wood: I am certainly deeply interested in this potassium sulphocyanate theory, as I suppose any of us who have attempted to treat pyorrhea and had to buck up against a case now and then where the local treatment does not seem to do much good. I think we are all of us looking for something for that rare case, perhaps one case in a hundred, where local treatment does not seem to suffice entirely, and where we do not seem to be satisfied with the results of our most careful treatment. I have had some patients where I have prescribed Lithia Salts, and have them return in a month with, so far as I could see, very marked improvement over what I was able to get with the local treatment. Now I have been such a thorough believer in the local treatment alone that I have been very backward to say anything of this sort. I did not like to admit it, and yet this occasional case seems to demonstrate that fact.

We all see mouths occasionally where they seem to be absolutely free from caries, the teeth are very easily kept in perfect order, and it seems to me very possible that this must be the mouth that has a saliva with the restraining element of potassium sulfocyanate in solution. But for the present, for me at least, I am very well satisfied to push the prophylaxis treatment for the sake of the benefits of a good clean mouth, whether it stops caries or not, and whether it absolutely cures for all time pyorrhea or not; just for the satisfaction of the patient and myself in producing and keeping clean mouths, I most heartily advocate and urge in every way the prophylaxis treatment for every case.

I had a well educated gentleman, who is very much interested in a benevolent work, where my son is also interested, and whom I believe is doing a grand work, call to see me in regard to the work he is doing. He sat down at my desk and talked with me for a few minutes and showed me some pictures. Because of our necessary close proximity, I got the odor from his mouth and the sight of his teeth. I immediately lost all interest in the man's work. I had to turn my head immediately, and while I still thought well of the gentleman, I was obliged to tell my boy, when I went home, that if that man would just call on his dentist and get a proper treatment for his teeth and mouth, he would accomplish a great deal more in his work. Now we meet these cases right along every day, people with fine characters and in every way good citizens, but, alas, a foul mouth.

Such conditions surely discredit the standing in the community of any man who will tolerate such a condition. So I say I shall pound away on the prophylaxis end of it just for that feature alone, a clean mouth, and I know from my own experience with many of my own patients, that whereas they were in the habit of coming back every year, after having a general fix up, to get fixed up over again, with a few cavities here and there, now with a regular monthly or bi-monthly treatment, whichever the case demands, they don't have these cavities. In many instances they drop about 75

percent. of their carious cavities. They don't develop them because of the vigorous use of the floss and the brush and tooth-powder which they intelligently use. It is impossible to get them all to use the toilet intelligently, but we must keep at them. I have such results so far that I have no question but that I can combat any case of pyorrhoea successfully. There is that occasional case, one in a hundred, that seems to be benefited by systemic treatment, but only occasionally.

Dr. Bowles: Most of the matter in the paper I have read to you is not mine, so I cannot defend it; it is work of the leaders in our profession, as expressed in our current literature—I simply made abstracts of these papers for your consideration.

I want to say for myself, however, that systemic treatmet is simply a wider and broader prophylaxis. The orange wood stick is an adjunct, but I believe it to be a minor force. I believe this sulphocyanate will widen prophylaxis to an extent equal to that which Dr. Smith has made for his orange wood stick. You remember he made the statement, when he was here, that he could arrest 90 percent. of all caries. I notice he has modified that somewhat in some of his later talks before dental societies, to 75 percent. Dr. Wood and Dr. Rogers and some other expert wielders of the orange wood stick may have equal success, but I cannot always get it. I cannot cure 90 percent. of pyorrhoea cases as Dr. Wood can; that occasional incurable case with respect to me, is more likely to be 25 percent. I believe that if I could keep the teeth clean for 24 hours every day, that I could prevent caries, but cannot do it with the orange wood stick or the floss or tooth-brush, in the hands of my patients. I believe that it is absolutely impossible for me to thoroughly clean my own teeth, or for any patient to thoroughly clean his. We know that the forces breaking down the teeth are working 24 hours a day, when the bacteria are promulgating rapidly, and the teeth are literally almost being melted down. If we could get our remedies into the system which will be working away for us with the bacteria, for 24 hours every day, on the bacteria itself, then we have a force in conjunction with the orange wood stick which ought to be invaluable.

I have been making some tests, and one thing I seem to have proven to my own satisfaction, and that is that where the teeth are melting down there is certainly an absence of the potassium sulfocyanate. You can all make this test easily. All you have to do is to take two c.c.m., (32.2 drops) of saliva and mix it with an equal amount of distilled water, shake it thoroughly in the test tube, and to that add five drops of per chloride of iron, and if there is a sufficient amount of potassium sulphocyanate present to render the tooth safe there will appear a deep wine color stain which will shade down to a lemon canary. If you wish to make a control, simply put your per-chloride of iron in the distilled water, and you will get the lemon color.

While, as I say, I have found that it works almost invariably, to-day I have had my first exception. A patient came in to-day whose teeth are going rapidly, and I asked her to give me some of the saliva. We worked for about half an hour. I got about sixteen drops, hardly enough to make a test, but with what I did have the color seemed to show up well, but I suspect that perhaps one reason why caries is going on rapidly there is that the saliva is so scant that its action as a neutralizing agent is almost nothing to speak of. Where teeth are immune the coloring is very marked, and vice versa. Now I have a number of patients on the sulphocyanate treatment. As Dr. Graham says, I cannot demonstrate anything specific, but I am demonstrating a number of things to my own satisfaction. One case, a young girl, who came to me about six months ago, mouth in a foul condition. In the first place I had to go over her teeth with the orange wood stick and pumice, and then I failed to remove all the deep-seated stains. I even had to use the tube brush a good deal and the orange wood point in the engine and polish for a long time and vigorously. Each time that girl came to me (she came over a period of four or five months) I had to polish those teeth. I finally got some of the sulphocyanate tablets from Parke, Davis & Co. and put her on a half grain tablet for three weeks, when her saliva tested equal to that of an immune. This girl's saliva was changed so that its reaction was all right, and while, of course, one case of this kind does not prove anything, yet in the three weeks during which she has been taking these tablets, her teeth are certainly free from deposits, and during the past three weeks, and I will see her again tomorrow, which will make the fifth week that she has been under my treatment, her teeth certainly have been cleaner, and are easier to keep clean. While this does not prove much, it is at least encouraging.

